# Effect of 12 Weeks Core Training on Core Muscle Performance in Rhythmic Gymnastics

**DOI:** 10.3390/biology10111210

**Published:** 2021-11-19

**Authors:** Paula Esteban-García, José Fernando Jiménez-Díaz, Javier Abián-Vicén, Alfredo Bravo-Sánchez, Jacobo Á. Rubio-Arias

**Affiliations:** 1 Performance and Sport Rehabilitation Laboratory, PerlaSport Group, Faculty of Physical Activity and Sport Science, University of Castilla la Mancha, 45071 Toledo, Spain; grupo.perlabsport@uclm.es (J.F.J.-D.); javier.abian@uclm.es (J.A.-V.); alfredo.bravo@uclm.es (A.B.-S.); 2 Department of Education, University of Almeria, 04120 Almería, Spain; jararias@ual.es

**Keywords:** strength, muscular activity, electromyography, core endurance test, muscular performance

## Abstract

**Simple Summary:**

The aim of this study was to analyze the effect of 12 weeks of core muscle training on core muscle performance in rhythmic gymnasts. Core strength training leads to improvements in body composition, as well as improvements in trunk strength and increases in muscle electromyographic activity. These improvements could therefore improve performance during competitive rhythmic gymnastics exercises.

**Abstract:**

Background: Rhythmic gymnastics performance is characterized by technical elements involving flexibility, aerobic capacity and strength. Increased core strength in rhythmic gymnastics could lead to improved sporting performance. Objective: The aim of this study was to analyze the effect of 12 weeks of core muscle training on core muscle performance in rhythmic gymnasts. Methods: A randomized controlled study involving 24 rhythmic gymnastics was conducted. Participants were randomly assigned to a control group (CG; *n* = 12; age 13.50 ± 3.17 years) or a training group (TG; *n* = 12; age 14.41 ± 2.35 years). Body composition, isometric strength of trunk, core endurance and core muscle electromyographic activity were measured (EMG) after 12 weeks of core training. Independent sample t-tests were carried out to compare baseline values between groups. A two-way repeated-measures analysis of variance (ANOVA) (time × group) was applied. Results: The TG improved body composition, trunk lean mass (mean differences MD = −0.31; *p* = 0.040), lean mass (MD = 0.43; *p* = 0.037) and bone mass (MD = −0.06; *p* < 0.001) after training. Core training increased isometric strength of trunk, flexion test (MD = −21.53; *p* = 0.019) and extension test (MD = 22.7; *p* = 0.049), as well as the prone bridge core endurance test (MD = −11.27; *p* = 0.040). The EMG values also increased in the TG in prone bridge for front trunk (MD = −58.58; *p* = 0.026). Conclusions: Core strength training leads to improvements in body composition, as well as improvements in trunk strength and increases in muscle electromyographic activity. These improvements could therefore improve performance during competitive rhythmic gymnastics exercises.

## 1. Introduction

Rhythmic gymnastics started as a sport in the 1940s and debuted as an Olympic sport at the 1984 Olympic Games [1]. Aesthetic movements, flexibility, artistic and competitive components are distinct characteristics of rhythmic gymnasts [2]. Bobo-Arce and Méndez-Rial (2013) suggested that rhythmic gymnastics is a sport with a particular training process, very young athletes, earlier specialization, a large volume of training, lots of repetition and high levels of physical and psychological stress in competition. Elements of physical fitness such as flexibility, strength and aerobic capacity have been shown to be determinants of performance in rhythmic gymnastics [3,4]. Thus, physical, technical and psychological skills, and motor control and harmony of movement are key factors in the performance of gymnasts [2]. 

For appropriate control and harmony of movements, gymnasts need adequate strength development, which allows them to maintain technical elements of great amplitude. In gymnastic disciplines, to perform a maximum number of strength elements in a competition routine, a high level of specific strength endurance is required [5]. Relative strength is considered to be a more important determinant of gymnastics performance than absolute strength [6], which is why many training systems use the gymnasts’ own body weight to prepare them [7]. In this respect, an example of strength training with body weight is the training of the central trunk muscles (core). It is suggested that having a strong core allows for the complete transfer of forces developed with the lower extremities through the trunk to the upper extremities [7]. Many gymnastic movements are generated in the lower body, with the flexion-extension of the legs giving rise to positions held by the whole body for a few seconds, which require isometric and stabilizing strength of the central musculature, mainly. Therefore, an adequate development of the core in rhythmic gymnasts could evoke an increase in sporting performance [1], helping the execution and maintenance of technical movements. Furthermore, a link has been established between trunk stability and lower limb injuries or low back pain [8], so that specific trunk training could reduce this risk [9]. 

In order to be able to assess the force generated by gymnasts or athletes, there are quantitative measurements of maximal voluntary strength that can be performed with isometric testing on isokinetic dynamometers [9]. In these tests, maximum voluntary contraction (MVC) can be performed in both flexion and extension to quantify trunk strength [8]. On the other hand, for the measurement of endurance strength in athletes, trunk tests such as the McGill test are often used to assess endurance capacity and core stability [10]. Muscle activation assessment tests, such as surface electromyography (sEMG), can also be considered useful tools for assessing muscle activation [8]. In sport, the positive relationship between muscle activation and performance can be established [11]. 

On the other side, the study of anthropometric variables associated with sports performance is interesting because some studies associate variables such as weight, height, body mass index and lean mass with strength [12,13]. In gymnasts, a negative relationship has been established between fat mass values and improvements in strength and performance [14]; this makes it interesting to assess the gymnasts’ body composition and its possible relation to training.

In some sports, improving trunk strength and endurance can increase the ability to generate and maintain strength [15]. Demand for athletic performance responses by the muscles of the whole body and core acts as a bridge between the upper and lower extremities and provides a stable base to transfer force to the extremities [16]. Strength and endurance training of the core musculature could increase trunk stability in gymnasts, facilitating the transmission of forces generated between the upper and lower limbs [16,17]. Furthermore, it has been shown that the improvement in trunk strength is positively related to the extensor strength of this musculature, allowing gymnasts to achieve greater technical performance in all their back trunk extension movements [18].

Several studies suggest that athletes should perform trunk strength training to improve their athletic performance [10,12], demonstrating the effect of trunk training on athletes’ performance. However, there are few studies that analyze the effect of specific trunk training in rhythmic gymnasts on trunk muscle performance. Considering that specific trunk training, in addition to rhythmic gymnastics training, could improve trunk strength and stability and thus indirectly improve performance, the aim of this study was to analyze the effect of 12 weeks of core training in gymnasts who were still training in rhythmic gymnastics on body composition, isometric trunk strength, trunk endurance and electromyographic activity of trunk muscles.

## 2. Materials and Methods

### 2.1. Study Design

This study used a randomized, controlled single-blind design. A quasi-experimental intra- and inter-subject design with pre- and post-test, and with a control group, was used to identify the effects of 12 weeks of core training on the performance of the core muscles. Subjects were randomized into two groups: a control group (CG) or a training group (TG).

### 2.2. Participants

A total of 24 national women rhythmic gymnasts (*n* = 24; age 13.95 ± 2.77 years; height 151.39 ± 12.34 cm; weight 43.00 ± 12.82 Kg) were randomly divided into two groups: CG (*n* = 12; age 13.50 ± 3.17 years; height 147.87 ± 11.63 cm; weight 38.76 ± 11.91 Kg) and TG (*n* = 12; age 14.41 ± 2.35 years; height 154.91 ± 12.50 cm; weight 47.25 ± 12.74 Kg). The gymnasts of both groups continued their rhythmic gymnastics training on a regular basis, and core training was only applied to the gymnasts of the TG group. All participating gymnasts followed the same training, both gymnastic and core specific. The training protocols (gymnastics and core) were designed by the study researchers and subsequently applied by the trainers, previous familiarization and an informative session. In order to ensure the process, the study’s principal investigator monitored the training sessions. The inclusion criteria were that they had training experience of 2 years, competed in the national category and trained ≥9 h per week. All the gymnasts and their parents received written and verbal information regarding the nature of this investigation and provided written informed consent before the beginning of the study. Ethical approval was obtained from the Clinical Research Ethics Committee of the Toledo Healthcare Area (number 112/2015). This study complied with the ethical principles of the Declaration of Helsinki.

### 2.3. Procedures

The week before the start of the measurements, the gymnasts performed a 90 s warm-up and then were familiarized with the isometric and core endurance tests at moderate intensity, and in addition, signed the informed consent documents. On the day of data collection all the measurements were taken by the authors and the instruments were calibrated prior to use. First of all, stature and body mass were measured on a portable scale with a stadiometer (model 700, Seca, Hamburg, Germany) and body composition and densitometry were recorded. Then the rhythmic gymnasts completed a 10 min warm-up on a bicycle ergometer, using self-chosen resistance at 40–60 rpm (20–30 watts), followed by 5 min of stretching exercises for the trunk and lower extremities, the isometric test, and McGill’s core endurance test. Surface electromyography (sEMG) of the core was recorded during the isometric and McGill’s core endurance tests (Table 1). 

Body composition and densitometry measurements were taken following the standardized techniques of the International Society for the Advancement of Kinanthropometry (ISAK), fat mass (FM, in Kg) (ICC: 0.99–0.98; CV: 2.6%), total lean mass (LM, in Kg) (ICC: 0.99–0.99; CV: 0.8%), bone mass (BM, in Kg) (ICC: 0.99–0.99; CV: 0.6%) fat tissue percentage (FT%) (ICC: 0.99–0.99; CV: 2.7%) and trunk lean mass (TLM, in Kg) (ICC: 0.99–0.98; CV: 1.6%) were assessed using dual-energy X-ray absorptiometry (DXA) (Lunar iDXA, General Electric Healthcare, Fairfield, CT, USA) [19]. 

**Figure 1:**
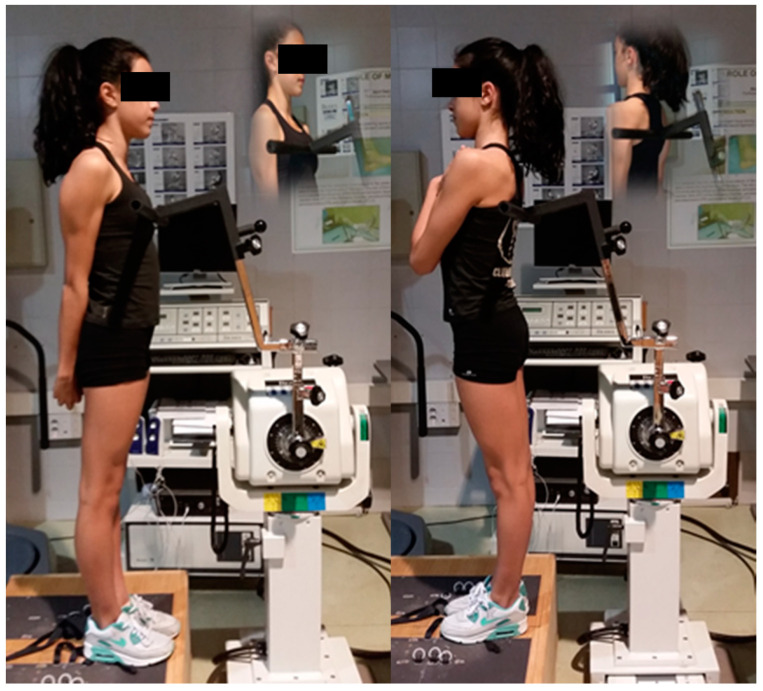
Isometric strength measurements.

The isometric tests for maximum strength of trunk were performed with a Biodex isokinetic dynamometer (Biodex System 3; Biodex Medical Systems, Inc., Shirley, NY, USA). Maximum voluntary contraction (MVC) exerted in isometric contraction for trunk flexion and extension was evaluated in terms of peak torque (PT, in N·m) (ICC: 0.87–0.92; CV: 10.5%). Isometric strength measurements were made following the protocols described by Waldhelm and Li (2012) [20] (Figure 1). Trunk flexion and extension were performed while standing, with trunk straight, looking straight ahead, pelvis stabilized, and without upper extremity support. The average of three peak torque with 2 min rest in between was taken for later analysis. The gymnasts held each contraction for 5 s with 30 s rest between trials [19]. 

Core endurance was measured for the same person with the McGill test [10]. The core endurance tests were the extensor endurance test or Biering-Sorensen test (Sorensen) and the prone bridge test (prone bridge). Gymnasts maintained these positions as long as possible, and the time was measured in each test in s. Both tests were considered failures when the gymnast lost the horizontal with respect to the floor. The Sorensen test began with lying prone, with the lower body manually fixed, hips extended over the edge of the test surface, and hands on the opposite shoulders. The prone bridge test was performed on the ground. The gymnasts had to maintain the prone position supporting themselves on their feet and forearms with shoulders and elbows in 90° flexion. Forearms needed to remain pronated. 

sEMG was measured during McGill’s core endurance and isometric tests. An 8-channel sEMG ME 6000TE (Mega Electronics, Kuopio, Finland) was used for data collection. sEMG signals from the flexor muscles of the front trunk were analyzed as a group, as were the extensor muscles of the back trunk. The average value of muscle activation (EMG root mean square (rms), EMG_rms_ in µV) (ICC: 0.87–0.94; CV: 12.8%) was measured during the middle 3 s of the 5 s of contraction. Each gymnast’s skin was prepared for sEMG evaluation according to guidelines of the SENIAM organisation [21], including scrubbing and cleaning with alcohol. Electrodes were placed bilaterally on the front trunk muscles (rectus abdominis, external oblique abdominis) and back trunk muscles (erector spinae). Two 10 mm diameter Ag-AgCl surface electrodes were used on each muscle for data collection. The sampling rate was set at 1000 Hz per channel. The signals were filtered at 500 Hz, and further filtered. The raw data were stored and subsequently processed. The sEMG data were fully rectified and smoothed and the rms was normalized to the signal recorded with peak maximum value [22].

### 2.4. Intervention

**Figure 2:**
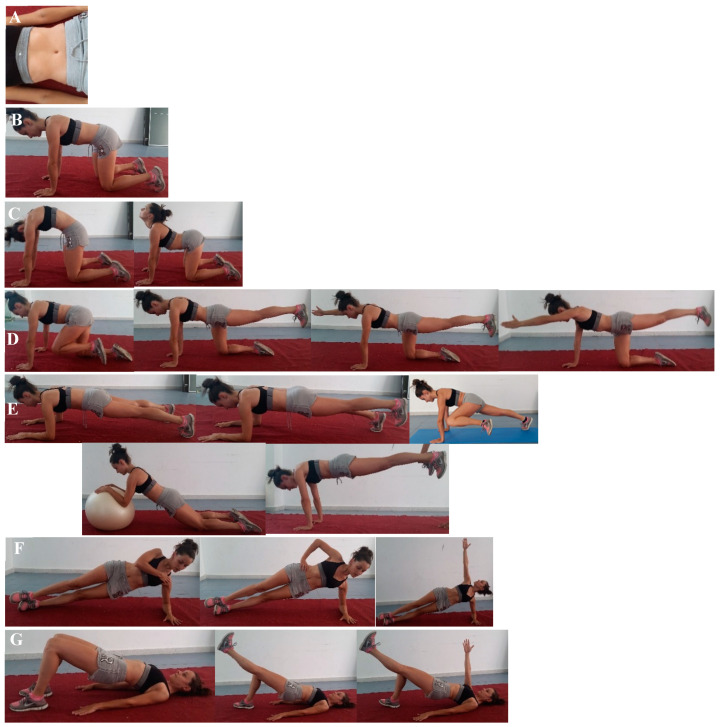
Core muscular training exercise. (**A**) Hollowing; (**B**) Bracing; (**C**) Dissociation; (**D**) Cat-Camel; (**D**) Quadrupedal; (**E**) Front Bridge; (**F**) Side Bridge; (**G**) Supine Bridge.

Core muscular training was performed in two alternative sessions per week for 12 weeks, supplementary to gymnastic training, included three progressions of difficulty, periods 1, 2, and 3 (Table 2) and each period lasted for 4 weeks. The core program was based on core training by McGill, (2010), increasing the number of series and not the maintenance time of the isometry, due to the commitment to the level of tissue oxygenation in this type of prolonged contraction [23]. The core exercises were performed at the end of the rhythmic gymnastics’ session. The core program comprised eight exercises, that is, hollowing (A), bracing (A), dissociation of shoulder girdle and pelvic girdle (B), Cat-Camel (C), quadrupedal stance (D), front bridge (E), side bridge (both sides) (F) and supine bridge (G) (Figure 2). 

### 2.5. Data Analysis

Statistical analysis of data was performed with the Statistical Package for the Social Sciences (IBM Corp. IBM SPSS Statistics for Windows, Version 24.0. Armonk, NY, USA: IBM Corp.). Descriptive statistics were calculated using the mean and standard deviation and the mean difference using confidence intervals. The Shapiro–Wilk test was used to analyze data distribution, getting a normal distribution. Subsequently, independent sample *t*-tests were carried out to compare baseline values between groups. In addition, a two-way repeated-measures analysis of variance (ANOVA) (time × group) was applied to analyze the effect of the intervention on outcomes. Eta squared (η^2^) effect sizes for the time × group interaction effects were calculated. An effect of η^2^ ≥ 0.01 indicates a small, ≥0.059 a medium, and ≥0.138 a large effect. For those variables that showed significant main effects, post-hoc tests (Bonferroni) were performed. The effect size (d) was calculated following the guidelines of Cohen [24]. The d was considered large (>0.80), moderate (0.5) and small (<0.2). An effect was considered statistically significant when *p* ≤ 0.05. 

## 3. Results

All participants completed the intervention and were included in the data analysis. No difference was observed between groups at baseline. Maximum growth velocity (MGA) was measured as a widely used indicator to assess biological maturation [25]. The age and height of the subjects were used to determine their biological maturation [26]. No significant differences in biological maturation were found between pre- and post-training in CG (*p* = 0.349), TG (*p* = 0.339) and between CG and TG in pre-training (*p* = 0.351).

### 3.1. Body Composition and Densitometry

Results for body composition are presented in Table 3. We observed no differences between the two groups for either of the two time-line measurements (*p* > 0.05). Within-group analysis showed an increase in the TG between pre- and post-core training in TLM (*p* = 0.040, d = −0.7; 95% confidence interval [CI] of the mean differences [MD] of the score = 0.03 Kg, 1.29 Kg), in LM (*p* = 0.037, d = −0.7; 95% CI of MD = 0.04 Kg, 1.30 Kg), and in BM (*p* < 0.001, d = −1.3; 95% CI of MD = 0.52 Kg, 2.09 Kg), and the CG showed an increase in BM (*p* = 0.003, d = −1.1; 95% CI of MD = 0.35 Kg, 1.79 Kg) and a decrease in the FT% (*p* = 0.044, d = 0.5; 95% CI of MD = −1.12%, −0.09 Kg). 

### 3.2. Isometric Tests in Isokinetic Dynamometer and Electromyography Analysis

Results for the PT and EMG_rms_ in the isometric tests are presented in Table 4. We observed no differences between the two groups for either of the two measurements (*p* > 0.05). Within-group analysis of the TG showed increases (*p* < 0.05) between pre- and post-core training in PT in the flexion isometric test (*p* = 0.019, d = 0.6; 95% CI of MD = 0.03 N·m, 1.20 N·m) and the extension isometric test (*p* = 0.049, d = 0.5; 95% CI of MD = 0.07 N·m, 1.15 N·m). In addition, the CG showed decreases of EMG_rms_ in front trunk in the flexion isometric test (*p* = 0.03, d = 0.6; 95% CI of MD = −1.19 µV, −0.04 µV) and the TG decreases of EMG_rms_ in the back trunk in the extension isometric test (*p* = 0.04, d = 0.7; 95% CI of MD = −1.326 µV, −0.054 µV).

### 3.3. Endurance Test and Electromyography Analysis

**Figure 3:**
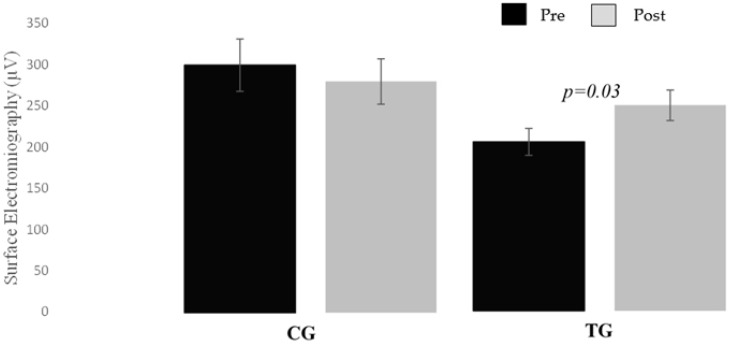
Significant difference EMG_rms_ front in prone bridge endurance test/TG.

Results for the core endurance test are presented in Table 5. We observed no differences between the two groups for either of the two endurance core tests (*p* > 0.05). Within-group analysis of the TG showed an increase between pre- and post-training in prone bridge (*p* = 0.044, d = −0.5; 95% CI of MD = 0.083 s, 1.131 s). For EMG in the endurance test, we observed no differences between the two groups for either of the two tests (*p* > 0.05). However, within-group analysis of the TG showed an increase between pre- and post-core training in EMG_rms_ front trunk in prone bridge (*p* = 0.030, d = −0.5; 95% CI of MD = 0.035 µV, 1.197 µV) (Figure 3).

## 4. Discussion

The aim of this study was to analyze the effect of 12 weeks of core training in gymnasts who were still training in rhythmic gymnastics on body composition, isometric and endurance strength core and core muscle electromyographic activity. The main findings were that the core training evoked an increase in trunk lean mass, lean mass and bone mass, and moreover the values of isometric strength and endurance strength and EMG in the core during the endurance test improved. 

Regarding body composition, the TG showed higher values of TLM, LM and BM after core muscular training and the CG in the BM and lower values in the FT%. To our knowledge, there are no studies on the effect of core training on the body composition of gymnasts. However, it is possible to find similarities with our results in the study by Skrypnik et al. [27], where different types of interventions, resistance training and endurance strength training were compared on body composition. Only the resistance strength training group obtained a significant increase in total lean body mass (<0.001) and total fat-free body mass (<0.001). In this respect, therefore, the gains in lean mass with resistance training, used in core muscle training, would be justified. Similarly, Piacentini et al. [28], evaluated the effects of two different strength training protocols on resting metabolic rate, body composition, running economy and strength parameters, in young elite endurance athletes. Both training protocols included core muscle strength, and both also showed a decrease in body fat percentage and fat mass that reflected a significant increase in fat-free mass in the young athletes. On the evidence of these results, it can be said that the changes in body composition produced by core training in gymnasts may be due to the influence that strength training has on these parameters. In addition, the CG showed lower fat mass values after the intervention period, which can be explained by higher initial fat mass values from this group and by the CG continued with their usual gymnastic training, the effect of rhythmic gymnastics training cannot be ruled out. The effect that gymnastic training has on athletes in increasing bone mass has been demonstrated in comparison to other sports or control subjects [29,30]. This is related to the fact that both training groups in our research showed significant increases in BM. This is because the subjects were 13.95 ± 2.77 years old and in puberty, when bone mass mineral accrual increases substantially during the growing years [31]. Puberty is an opportune time for bone strengthening [32], when the mechanical loading of athletic training is a positive factor for skeletal strength, for maximizing bone mineral gain and reducing the risk of osteoporosis in later life [33,34]. Gruodyte-Raciene et al. [35], and Gruodyté et al. [30], consider that gymnastic training is especially osteogenic for bone development in children and adolescents. Therefore, although gymnastic training may already have a positive effect on the body composition of gymnasts, added core training could have greater benefits for the body composition of female athletes. A relationship is established between gymnasts’ body composition and performance, with low values of fat mass being a determinant of performance [14,36].

In relation to isometric strength in the isometric test on the dynamometer, significant effects were found between pre- and post-core training in the TG. There are no studies on rhythmic gymnastics or other sports about the effect of core training on isometric trunk strength. Improvements in isometric trunk strength, both in flexion and extension, of gymnasts after training benefit these athletes, because they need upper body endurance strength and trunk muscle function to be successful in competition. Improving trunk strength and endurance would allow gymnasts to increase their ability to generate and maintain force throughout their routine. Core stability might contribute to the gymnast’s performance as it would facilitate the transmission of forces generated by the lower to the upper body during technical elements and it would enhance balance control [15]. The positive data on the gymnasts’ isometric strength after core training could reflect the positive effect of core training as a complementary training to gymnastic training. On the other hand, the results obtained in muscle activation, during the isometric test on the dynamometer, reflect a decrease in both study groups. This may be due to other types of neural adaptations that are not evaluated with the amplitude of the sEMG signal, such as inhibition of the antagonistic muscles, greater activation of the synergistic muscles or better inter-muscular coordination [37].

Similarly, the results obtained in the McGill endurance test and muscle activation in these tests, reflected significant effects between pre- and post-core training. The TG rhythmic gymnasts increased the maintenance time in prone bridge, as well as the muscle activation in the front trunk. In accordance with these results, previous studies have demonstrated that core training increases the maintenance time in the endurance test, and so increases trunk strength and stability strength in women collegiate gymnasts [38], dance students [39] or competitive collegiate dancers [40]. In this sense, the added and positive effect that core training could have on the gymnasts is again reflected.

Several considerations and limitations should be acknowledged. The evaluation of performance in rhythmic gymnastics was not carried out, so it cannot be confirmed that improvements in the training group had a direct influence on performance in competition. There was no control of the external activities that the participants of the sample did outside of the training. The sample size of the study can be considered small. However, the study has the strength to be considered the first to evaluate the effect of core training on national level rhythmic gymnasts. This core training program considered that the improvements found in the gymnasts are due more to core training combined with gymnastic training than to rhythmic gymnastics training alone because some improvements only occurred in the group that performed core training. Therefore, possible lines of research could analyze the effect of this type of core training on gymnastic performance, on the execution of technical gestures or on the judges’ evaluation.

## 5. Conclusions

Our results suggest that combining a traditional rhythmic gymnastics program with a core training program could lead to increased strength and improved body composition. Additionally, core strength training produces improvements in trunk strength values in gymnasts, in addition to increasing muscle activation values. 

## 6. Practical Applications

The proposed training is considered a useful tool for the training of gymnasts by their coaches. The improvements observed in the group that carried out a core program in addition to their traditional training presented improvements in strength and muscle activation capacity and this could have a positive transfer to competition. However, more studies analyzing the transference effect towards competition are needed.

The gains in strength and stability achieved will help coaches improve the physical preparation of gymnasts, and thus increase the technical level.

In addition, core muscle strength training may be of interest to another type of population, such as older adults, since ageing is associated with a variety of biological changes that can contribute to the decline of skeletal muscle mass, strength, and function [41].

## Figures and Tables

**Table 1:** Study protocol.

Pre-Training	Core Training 12 Weeks	Post Training
Body composition and densitometry analysis		Body composition and densitometry analysis
Isometric test with EMG		Isometric test
Core endurance test EMG		Core endurance test

**Table 2:** The Core program.

Exercises	Period 1	Period 2	Period 3	
	Volume	Volume	Progress	Volume
Hollowing	10 sets	10 sets		
Bracing	10 sets	10 sets		
Dissociation	5 sets	5 sets		
Cat-Camel	10 sets	10 sets	Supine Bridge	2 × 5 sets × 20 s (15 s rest) (both legs)
Quadrupedal	8 sets of 20 s (15 s rest)	2 × 7 sets of 20 s (15 s rest)	Quadrupedal Birddog exercise	2 × 5 sets
Front Bridge			Front Bridge	2 × 5 sets (both sides)
			Front Bridge destabilisation	2 × 5 sets × 20 s) (15 s rest)
			Front Bridge on swiss ball	2 × 5 sets × 20 s) (15 s rest)
Side Bridge			Side Bridge	2 × 5 sets × 20 s (15 s rest) (both sides)
Supine Bridge			Supine Bridge	2 × 5 sets × 20 s (15 s rest) (both legs)

**Table 3:** Body composition and densitometry results (Mean ± SD).

	Control Group (*n* = 12)				Training Group (*n* = 12)				
	Pre-Training	Post-Training	Mean Differences	*p*	Pre-Training	Post-Training	Mean Differences	*p*	Interaction Time × Group (*p*)
FM (kg)	8.74 ± 3.47	8.54 ± 3.51	0.20	*0.165*	10.41 ± 3.66	10.57 ± 3.63	−0.16	*0.365*	*0.04 (0.85)*
LM (Kg)	28.84 ± 8.58	28.89 ± 8.00	−0.43	*0.793*	34.71 ± 7.94	35.14 ± 7.89	−0.43	*0.037*	*1.83 (0.19)*
BM (Kg)	1.65 ± 0.53	1.69 ± 0.54	−0.04	*0.003*	2.04 ± 0.60	2.09 ± 0.59	−0.06	*<0.001*	*0.72 (0.41)*
%FT (%)	23.10 ± 4.69	22.47 ± 4.64	0.63	*0.044*	22.67 ± 2.73	22.71 ± 2.79	−0.04	*0.856*	*2.60 (0.12)*
TLM (Kg)	13.68 ± 4.39	13.73 ± 4.18	−0.05	*0.669*	16.79 ± 3.92	17.10 ± 4.10	−0.31	*0.040*	*2.29 (0.12)*

FM: fat mass; LM: lean mass; BM: bone mass; %FT: average fat tissue; TLM: trunk lean mass; SD: standard deviation; *p* ≤ 0.005.

**Table 4:** Performance in isometric test and electromyography values.

	Control Group (*n* = 12)					Training Group (*n* = 12)				
		Pre-Training	Post-Training	Mean Differences	*p*	Pre-Training	Post-Training	Mean Differences	*p*	Interaction Time × Group (*p*)
Flexion test	PT (N·m)	26.52 ± 11.26	41.78 ± 27.05	−15.26	0.086	31.56 ± 12.39	53.09 ± 41.36	−21.53	0.019	0.27 (0.61)
	EMG_rms_ Front (µV)	390.92 ± 254.19	256.58 ± 135.61	134.33	0.03	386.33 ± 205.00	345.92 ± 217.18	40.42	0.494	1.30 (0.27)
	EMG_rms_ Back (µV)	45.08 ± 34.27	57.17 ± 43.17	−12.08	0.492	66.92 ± 35.42	86.42 ± 61.21	−17.29	0.272	0.09 (0.77)
Extension test	PT (N·m)	31.75 ± 17.28	39.44 ± 34.00	−7.69	0.444	40.89 ± 19.28	63.66 ± 53.36	−22.77	0.049	0.95 (0.34)
	EMG_rms_ Front (µV)	129.58 ± 73.85	178.00 ± 155.46	−48.42	0.199	207.25 ± 123.68	232.42 ± 165.36	−25.17	0.498	0.20 (0.66)
	EMG_rms_ Back (µV)	128.83 ± 94.92	104.42 ± 41.28	24.42	0.421	163.67 ± 106.92	98.67 ± 36.61	65.00	0.04	0.93 (0.35)

PT: peak torque; EMG_rms_: average electromyography activity; SD: standard deviation; *p* ≤ 0.005.

**Table 5:** Performance in McGill test.

	Control Group (*n* = 12)				Training Group (*n* = 12)				
	Pre-Training	Post-Training	Mean Differences	*p*	Pre-Training	Post-Training	Mean Differences	*p*	Interaction Time × Group (*p*)
Sorensen	32.57 ± 11.53	34.02 ± 22.32	−1.44	0.797	37.94 ± 19.86	51.00 ± 22.51	−13.06	0.172	1.23 (0.28)
Prone bridge	31.06 ± 16.57	24.74 ± 15.36	6.32	0.133	27.99 ± 13.86	39.26 ± 23.38	−11.27	0.04	5.92 (0.49)

SD: standard deviation; *p* ≤ 0.005.

## Data Availability

Not applicable.
